# A Saudi Hospital’s Experience in the Management of Well-Appearing Neonates at Increased Risk for Early-Onset Bacterial Sepsis

**DOI:** 10.7759/cureus.49570

**Published:** 2023-11-28

**Authors:** Sameer Y Al-Abdi, Abbas M Al-Omran, Naglaa I Moustafa, Zakyia S Al-Qoweri, Shaimaa A El-Nokbasy

**Affiliations:** 1 Pediatrics/Neonatology, Al-Ahsa Hospital, Al-Ahsa, SAU; 2 Pediatrics/Neonatology, Almana General Hospital, Al-Ahsa, SAU; 3 Pediatrics, Almana General Hospital, Al-Ahsa, SAU

**Keywords:** complete blood count, c-reactive protein, premature rupture of membranes, group b streptococcus, antibiotics, blood culture, neonate, early-onset sepsis

## Abstract

Background: Early-onset neonatal bacterial sepsis (EOS) is a serious medical condition where pathogenic bacterial species are isolated from the blood of newborns within the first 72 hours of life. Neonatal healthcare providers face challenges in managing well-appearing newborns born at 35 weeks gestational age or more who are at an increased risk of developing EOS. The American Academy of Pediatrics (AAP) has recommended three approaches for managing EOS. One of these approaches includes enhanced observation to observe the progression of the newborn's clinical condition within the first 48 hours after birth. The AAP recommends that birth centers should adopt institutional approaches that are tailored to their specific local resources and structures. It recommends that the chosen approach is evaluated to identify infrequent negative outcomes and to confirm its effectiveness.

Aims: To report our experience in managing EOS in newborns born at 35 weeks gestation or later with an increased risk for EOS.

Methods: This was a review of electronic medical records from the past five years. We included a sample of newborns born at or after 35 weeks gestational age who were at increased risk of EOS and appeared to be healthy. We implemented universal antenatal culture-based screening for Group B streptococcus (GBS). We followed the recommendations of the AAP in 2012 to manage these newborns. We performed a complete blood count (CBC) with differential and C-reactive protein (CRP) tests to predict EOS. We also considered the newborns symptomatic if they displayed any clinical signs of EOS.

Results: A total of 806 newborns were included in the study, out of which 27 (3.3%) of them had symptoms of EOS, while the remaining 782 newborns appeared healthy. Predictive blood tests were performed on 281 (34.9%) of the newborns, out of which 126 (44.8%) of them had a positive test result. However, blood cultures were obtained from 134 (16.6%) of the total cohort. Intravenous antibiotics were administered to 33 (4.1%) of the newborns. All symptomatic newborns had a positive predictive blood test result, and two of them had culture-proven EOS. Blood cultures obtained from the remaining 107 asymptomatic newborns were negative. In this context, 140 newborns needed to be pricked for positive predictive blood tests to predict one case of EOS. However, if the positive predictive blood tests were only performed on symptomatic newborns, then only 14 newborns would need to be pricked to predict one case of EOS.

Conclusion: Based on the present study, it is advised to follow the current AAP recommendation against predicting EOS by solely relying on CBC with differential or CRP. The study suggests that the enhanced observation approach is a more sensible option for managing EOS, but this needs to be confirmed in a larger study.

## Introduction

Early-onset neonatal bacterial sepsis (EOS) is a serious medical condition where pathogenic bacterial species are isolated from the blood of newborns within the first 72 hours of life [1-3]. Neonatal healthcare providers face challenges in managing well-appearing newborns born at 35 weeks gestational age or more who are at an increased risk of developing EOS [4]. One reason for this challenge is that although the incidence of EOS is rare, its associated morbidity and mortality are substantial [1,3,5]. A second reason for this challenge is that the currently available predictive laboratory blood tests for EOS are unreliable [4,5]. In addition, how EOS is managed can significantly impact maternal-neonatal bonding, and exclusive breastfeeding, and lead to various long-term health issues due to early antibiotic use [4].

The risk of EOS increases when mothers are colonized by group B streptococcus (GBS) in their gastrointestinal and genitourinary tracts, have ruptured amniotic membranes (ROM) more than 18 hours before birth, or have intra-amniotic infection (chorioamnionitis) [6]. The American Academy of Pediatrics (AAP) has recommended three approaches for managing EOS [6]. The first approach is called categorical risk assessment, which involves assigning a risk category to the baby based on maternal intrapartum temperature ≥38°C and administration of penicillin G, ampicillin, or cefazolin ≥4 hours before delivery. The second approach combines multivariate risk factor analysis and clinical condition evaluation using an EOS calculator. The third approach is to observe the progression of the newborn's clinical signs of illness within the first 48 hours after birth, which is known as enhanced observation. The AAP and others have recommended that birth centers should adopt institutional approaches that are tailored to their specific local resources and structures [5,6]. It has been recommended that the chosen approach be evaluated to identify infrequent negative outcomes and to confirm its effectiveness [3]. We aimed to report our experience in managing EOS in newborns born at ≥35 weeks gestational age with increased risk for EOS.

## Materials and methods

Management of newborns at risk for EOS at the studied hospital

We have implemented universal antenatal culture-based GBS screening at Almana General Hospital (AGH) in Al-Ahsa Governorate, Eastern Province, Saudi Arabia. We administered intrapartum antibiotic prophylaxis (IAP) for GBS-colonized mothers and mothers with ROM >18 hours before birth [7]. We considered IAP to be adequate if either penicillin G, ampicillin, or cefazolin were administered intravenously to the mothers at least four hours before delivery [7]. Conversely, we considered IAP to be inadequate if the administration of any of these antibiotics occurred less than four hours before delivery or if other antibiotics were administered instead. We considered maternal intrapartum temperature ≥38°C a proxy for intra-amniotic infection [7]. We considered inadequate indicated IAP or maternal intrapartum temperature ≥38°C as the two main EOS risk factors [7]. There was no rooming in policy in AGH. Thus, all well-appearing newborns born at ≥35 weeks gestational age were admitted to the well-newborn nursery (level I neonatal care) [8]. The care at our nursery includes recording vital signs, clinical status, and feeding patterns every four hours for all newborns.

**Figure 1:**
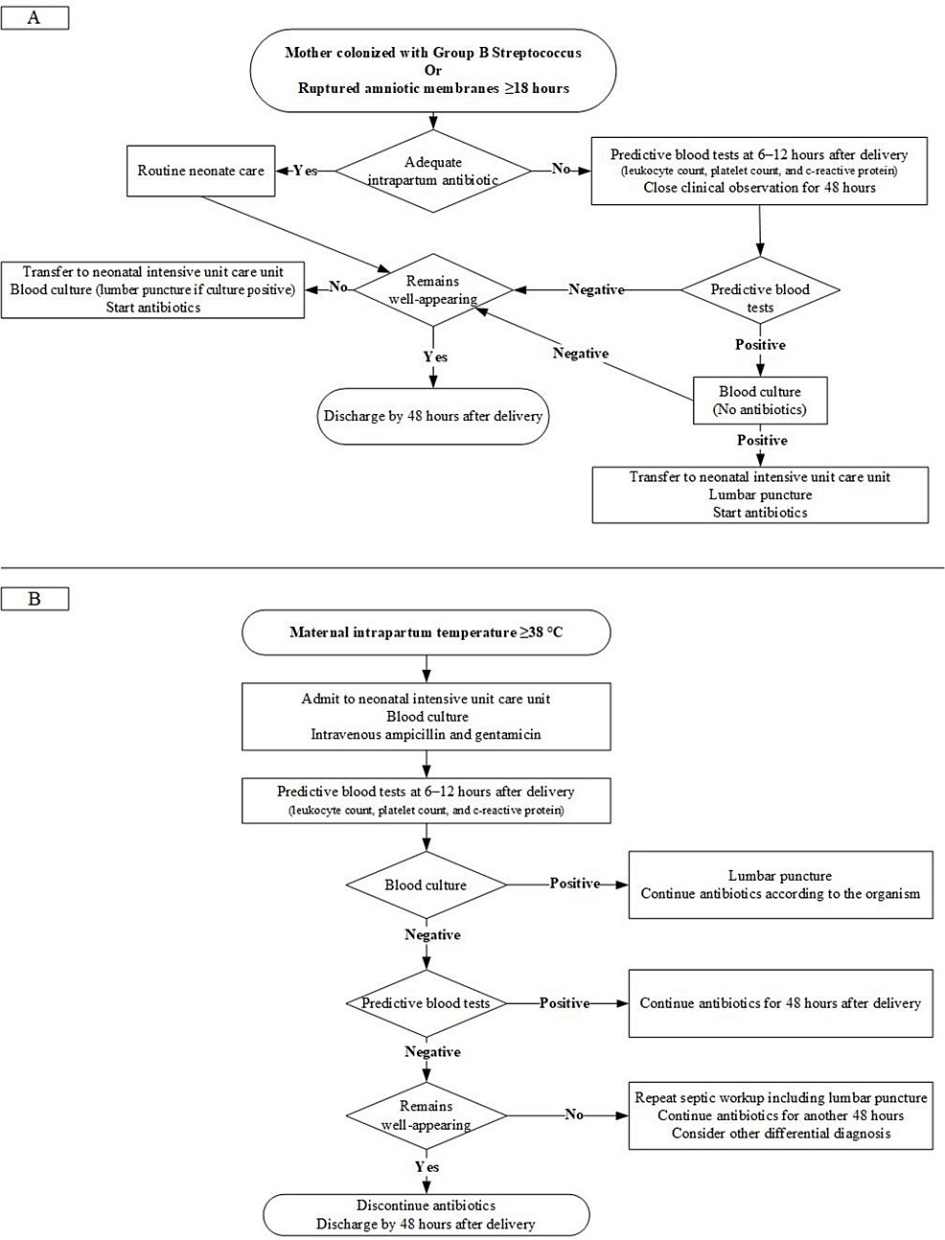
A summary algorithm for our management of newborns at risk for early neonatal sepsis

Figure 1 summarizes our management of newborns at risk for EOS. This management was based on recommendations of the AAP Committee on Fetus Newborns (COFN) in 2012 [7,9-11]. We consider blood culture to be the gold standard for EOS diagnosis [5]. We perform a complete blood count (CBC) with differential and C-reactive protein (CRP) at 6-12 hours after delivery to help predict EOS [5]. We consider the predictive blood tests positive if any of the following results were observed: Leukocyte count <5K/uL, immature to total neutrophil (I/T) ratio >0.22, platelet count <150 K/uL, or CRP >10 mg/L [7]. We considered the newborns symptomatic and may have EOS if they experience any of the following clinical indicators of possible EOS: temperature < 36°C or > 38°C, heart rate <100 or ≥160, mean blood pressure measurement less than gestation age in weeks, respiratory rate ≥60, respiratory distress, needs for respiratory support, apnea, seizure, decrease activity, feeding difficulties, feed intolerance, excessive gastric aspirates, abdominal distension, random blood sugar <46 mg/dL or >180 mg/dL without obvious cause, or base deficit of ≥10 mmol/L [2]. We administered ampicillin (50 mg/kg/dose every 8 hours) and gentamicin (4 mg/kg/dose every 24 hours) intravenously as the empirical treatment for EOS, and then we amended the antibiotics according to the blood culture sensitivity results.

Study design

This study was a retrospective electronic medical record review. We included a convenience sample of newborns born over five years, from 01 January 2016 through 31 December 2020. We included all well-appearing newborns born at ≥35 weeks gestational age with one or more of the EOS risk factors described above.

Statistical analysis

We presented categorical data as both numbers and percentages. Maternal age below 20 years, primigravida mother, and a 5-minute Apgar score below 7 may be associated with EOS according to previous studies [6,12-16]. Thus, we dichotomized these variables accordingly. We analyzed each twin individually. For the analysis, we used the Statistical Package for the Social Sciences (IBM SPSS Statistics for Windows, IBM Corp., Version 20.0, Armonk, NY).

## Results

**Figure 2:**
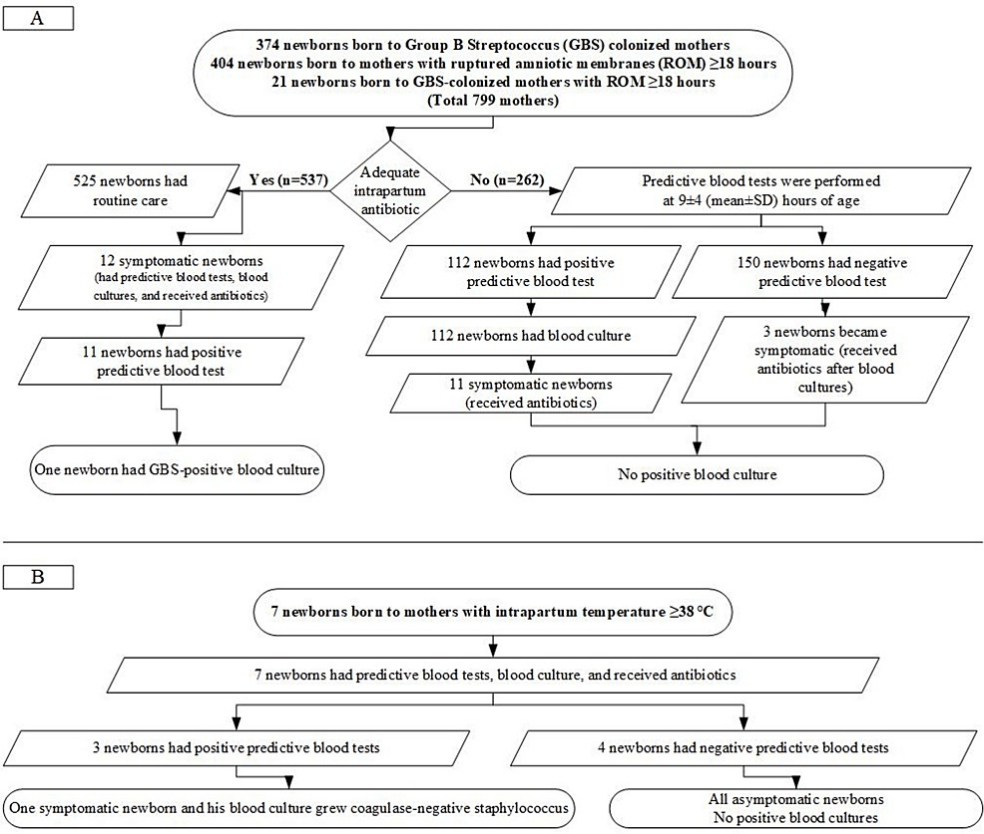
Summary of the results

During the five-year study period, 6,249 live births were recorded out of which 806 (12.9%) met the inclusion criteria for the study. Table 1 provides an overview of the basic characteristics of the included newborns and their mothers. Figure 2 summarizes the outcome of managing EOS in newborns who were at an increased risk of EOS and were born at or after 35 weeks of gestational age.

**Table 1:** The baseline characteristics of 806 newborns and their mothers who were included in the study.

Characteristics	Results
Gestational age (mean± standard deviation)	39.0±1.0 weeks
Birth weight (mean± standard deviation)	3098±420 grams
Number of male newborns	402 (49.9%)
Number of newborns with 5-minute Apgar score <7	zero
Maternal age (mean± standard deviation)	27±6.0 years
Number of mothers < 20 years old	79 (9.8%)
Number of primigravida mothers	264 (32.8%)
Number of cesarean section	187 (23.2%)

Among the 806 newborns included in the analysis, 281 (34.9%) infants underwent predictive blood tests for EOS. Among these 281 infants, 126 (44.8%) had positive results. However, only 16.6% (134/806) of the newborns in the study cohort had blood cultures performed, and only 4.1% (33/806) received intravenous antibiotics. Only 27 (3.3%) of the whole cohort showed symptoms of EOS, while the other 782 remained healthy. All the 27 symptomatic newborns had a positive predictive blood test. Among the symptomatic newborns with a positive predictive blood test, two had culture-proven EOS. Table 2 illustrates the basic, laboratory, and clinical features of these two newborns. Blood cultures obtained from the 107 asymptomatic newborns were negative. The study cohort did not have any confirmed cases of meningitis. In this context, 140 newborns were subjected to a positive predictive blood test to predict one case of EOS. However, if the positive predictive blood tests were performed only on symptomatic newborns, then 14 newborns would need to be tested to predict one case of EOS.

**Table 2:** Baseline, lab, and clinical characteristics of the two cases of culture-proven early neonatal sepsis

Characteristics	Newborn-1	Newborn-2
Maternal age	37 years	21 years
Maternal gravidity	6	2
Maternal health status	Gestational diabetes	Healthy
Mode of delivery	Normal vaginal delivery	Normal vaginal delivery
Early-onset sepsis risk factor	Ruptured amniotic membranes >18 hours	Intrapartum maternal temperature ≥38°C
Adequate intrapartum antibiotic prophylaxis	Yes	No
Gender	Male	Male
Gestational age	36 weeks	39 weeks
Birth weight	2550 grams	3550 grams
Symptoms	Poor feeding	Fever
Immature to total neutrophil ratio	0.21	0.62
Platelets count	139 K/uL	241 K/uL
C-reactive protein	5.0 mg/dL	12.6 mg/dL
Blood culture	Group B streptococcus	Coagulase-negative staphylococcus
Length of hospitalization	10 days	8 days
Clinical status at follow-up	Normal and healthy	Normal and healthy

Out of the 806 newborns that were analyzed, 653 (81.0%) were taken to the follow-up clinic within 28 days of their birth. Among these newborns, six were readmitted to the hospital due to having one or more clinical indicators of EOS. However, none of these infants had a confirmed infection except for a female newborn, who was diagnosed with an *Escherichia coli* urinary tract infection at 19 days old. Despite the urinary tract infection, the newborn remained in good health. The mother of this newborn was colonized with GBS and did not receive adequate IAP. This newborn was asymptomatic and had negative blood test results for EOS during her birth hospitalization.

## Discussion

The present study showed that 140 newborns born at ≥35 weeks gestational age with increased risk for EOS were pricked for predictive blood tests to predict one case of culture-proven EOS. But if these predictive blood tests were performed only on the symptomatic newborns, then only 14 newborns would be pricked to predict one case of EOS without missing true cases of EOS. These results support the current AAP COFN recommendation against predicting EOS by routine measurement of leukocyte count or CRP alone [6]. The two newborns who had a culture-proven EOS were among the symptomatic newborns and none of the well-appearing newborns had a culture-proven EOS. This indicates that the enhanced observation approach is a wise choice for managing EOS in newborns born at ≥35 weeks gestational age with increased risk for EOS.

The clinical condition of newborns within 24 hours of birth is a strong indicator of EOS as most newborns with EOS become symptomatic during this period [4,6,17]. However, using only the CBC and CRP to predict EOS is not reliable for well-appearing newborns born at ≥35 weeks gestational age [6]. Based on these facts, the AAP COFN supports using the clinical condition of the newborn to predict EOS and opposes relying solely on leukocyte counts or CRP levels [6]. Our results support this recommendation very well. Our study findings align with this recommendation, as none of the well-appearing newborns in our study had culture-proven EOS, and the two newborns who did have EOS were symptomatic. A previous study by Frymoyer et al. demonstrated that the enhanced observation approach was safe for over 20,000 newborns born at ≥35 weeks gestational age, regardless of their risk factors for EOS [17]. This approach resulted in a 60% overall decline in the use of CBC, CRP, and empirical antibiotics without missing any true cases of EOS [17]. However, it is important to note that this approach requires effort and the physical examination has limitations [5,17]. A recent retrospective study showed that using enhanced observation is safer than using the EOS calculator [18].

The most effective laboratory tests for predicting EOS and the acceptable number of newborns required to test and treat them remain unclear [2]. Predictive blood testing, such as CBC and CRP testing, requires blood samples, which are typically obtained through a heel prick or venipuncture. Unfortunately, these procedures can cause moderate pain in newborns [19]. Performing routine predictive blood testing on asymptomatic newborns at high risk for EOS can cause unnecessary pain and is not cost-effective. Therefore, it is essential to incorporate the principle of 'Choosing Wisely' to manage EOS and limit low-value testing to reduce costs and improve care quality [20].

According to the method of our study, it was not possible to calculate the rate of EOS at AGH. However, based on the EOS rate range (0.40 to 1.06 per 1,000 live births) reported in the other two nearby hospitals, it appears that the EOS rate at AGH would be on the lower end of this range. This is likely due to the universal antenatal culture-based GBS screening [21], which is carried out to prevent neonatal sepsis caused by GBS, the most common causative organism of EOS in the Arab states in the Gulf region [1]. Our two cases of culture-proven EOS were caused by GBS and CONS, which is the third most common causative organism of EOS in the same region [1].

In addition to being a retrospective study, several limitations of our study should be noted. Firstly, we did not have a follow-up on about 20% of the study cohort. Additionally, our total sample size is not large enough given the low EOS rate among the cohort. Moreover, due to the limited number of culture-proven EOS cases, we were unable to conduct a thorough statistical analysis to explore the potential risk factors associated with EOS in the study cohort. Lastly, while we considered maternal intrapartum temperature ≥38°C as a proxy for intra-amniotic infection, some professional organizations distinguish between isolated maternal intrapartum temperature between 38°C and 38.9°C and intra-amniotic infection [5].

## Conclusions

Based on the findings of the study, it was concluded that 140 newborns who were born at ≥35 weeks of gestation and had an increased risk for EOS would need to undergo predictive blood tests to detect one case of EOS. However, if these tests were conducted only on symptomatic newborns, then only 14 newborns would need to undergo the tests to detect one case of EOS without missing any true EOS cases. This implies that the enhanced observation approach is a more practical option for managing EOS. However, further research is needed to confirm this finding on a larger scale. It is recommended to follow the current AAP COFN recommendation against predicting EOS by solely relying on CBC with differential or CRP. It is important to abide by the principle of ‘Choosing Wisely’ in managing EOS.
